# Association between ventilatory ratio and mortality in patients with acute respiratory distress syndrome and COVID 19: A multicenter, retrospective cohort study

**DOI:** 10.1186/s12890-023-02733-9

**Published:** 2023-11-03

**Authors:** Henry M. Parada-Gereda, Janneth M. Avendaño, Johana E. Melo, Claudia I. Ruiz, Margarita I. Castañeda, Jorge Medina-Parra, Ricardo Merchán-Chaverra, Dinia Corzzo, Daniel Molano-Franco, Joan Ramón Masclans

**Affiliations:** 1 Intensive Care Unit Clínica Reina Sofia, Clínica Colsanitas. Clinical Nutrition and Rehabilitation Research Group, Fundación Universitaria Sanitas. Grupo Keralty, Bogotá, Colombia; 2 Intensive Care Unit Clínica Reina Sofia, Mujer y Pediátrica, Clínica Colsanitas, Clinical Nutrition and Rehabilitation Research Group, Fundación Universitaria Sanitas. Grupo Keralty, Bogotá, Colombia; 3 Intensive Care Unit Clinica Universitaria Colombia, Fundacion Universitaria Sanitas. Grupo Keralty, Bogotá, Colombia; 4 Department Clínica Reina Sofía, Clínica Reina Sofia, Mujer y Pediátrica. Grupo Keralty, Bogotá, Colombia; 5 Department Clínica Universitaria Colombia. Grupo Keralty, Bogotá, Colombia; 6 Clinical Nutrition and Rehabilitation Research Group, Fundación Universitaria Sanitas. grupo Keralty, Bogotá, Colombia; 7 Clinical Nutrition and Rehabilitation Research Group, Fundación Universitaria Sanitas. Clinica Santa Maria del Lago. Grupo Keralty, Bogota, Colombia; 8 Facultad de Medicina, Fundación Universitaria Sanitas, Bogotá, Colombia; 9 Latin American Nutrition Center (CELAN), Chía (Cundinamarca), Colombia; 10 Intensive Care Unit Clínica Reina Sofía, Intensive Care Unit Center of Cancer Research and Treatment (CTIC), Bogotá, Colombia; 11 Intensive Care Unit, Los Cobos Medical Center, Hospital San José, Center of Cancer Research and Treatment, Research Group Gribos, Bogotá, Colombia; 12 Critical Care Department, Hospital del Mar Barcelona, Barcelona, Spain; 13 Critical Care Illness Research Group (GREPAC), IMIM. Department of Medicine and Life Sciences (MELIS), Universitat Pompeu Fabra (UPF), Barcelona, Spain

**Keywords:** Artificial respiration, Ventilatory ratio, COVID-19, Respiratory distress syndrome, Mortality

## Abstract

**Background:**

Mortality rates in patients with COVID-19 undergoing mechanical ventilation in the intensive care unit are high. The causes of this mortality have been rigorously investigated. The aim of the present study is to establish mortality risk factors related to lung mechanics measured at days 1 and 5 in patients with covid-19 ARDS managed with invasive mechanical ventilation in the intensive care unit.

**Methods:**

A retrospective observational multicenter study including consecutive patients with a confirmed diagnosis of COVID-19-induced ARDS, admitted to three institutions and seven intensive care units in the city of Bogota between May 20, 2020 and May 30, 2022 who required mechanical ventilation for at least five days. Data were collected from the medical records of patients who met the inclusion criteria on day 1 and day 5 of mechanical ventilation. The primary outcome assessed was mortality at day 30.

**Results:**

A total of 533 consecutive patients admitted with ARDS with COVID-19 were included. Ventilatory ratio, plateau pressure and driving pressure measured on day 5 were significantly higher in non-survivors (p < 0.05). Overall, 30-day follow-up mortality was 48.8%. The increases between day 1 and day 5 in the ventilatory ratio (OR 1.42, 95%CI 1.03–2.01, p = 0.04), driving pressure (OR 1.56, 95%CI 1.10–2.22, p = 0.01); and finally plateau pressure (OR 1.9, 95%CI 1.34–2.69, p = 0.001) were associated with an increased risk of death. There was no association between deterioration of PaO_2_/F_I_O_2_ index and mortality (OR 1.34, 95%CI 0.96–1.56, p = 0.053).

**Conclusions:**

Ventilatory ratio, plateau pressure, driving pressure, and age were identified as independent risk factors for 30-day mortality in patients with ARDS due to COVID-19 on day 5 of invasive mechanical ventilation.

## Background

In most patients, the COVID-19 infection had a mild course with symptoms characterized by fever, loss of smell and malaise. However, 10–20% of patients developed severe disease requiring oxygen therapy and admission to the intensive care unit (ICU), progressing to acute respiratory distress syndrome (ARDS) and requiring mechanical ventilation (MV) [1]. Mortality in patients with critical COVID-19 was high, ranging from 15 to 74% when invasive MV was required [2].

Male patients who are active smokers and aged over 60 years face a higher risk of hospital death. Comorbidities including diabetes, arterial hypertension, cerebrovascular disease, respiratory diseases and chronic kidney disease also influence the prognosis of COVID-19 [3].

The ventilatory ratio (VR), calculated as [ventilation per minute (ml/min) × PaCO_2_ (mm Hg)]/(predicted body weight (kg) × 100 × 37.5), is a recently defined bedside measurement, which acts as a surrogate for the dead space fraction. It is easily obtained at the bedside with arterial blood gasometry and minute ventilation assessment [4]. It has been suggested that physiologic dead space is a stronger predictor of non-COVID 19 ARDS outcomes than oxygenation [5].

Monteiro et al. and Sinha et al. demonstrated that patients with ventilatory ratio > 2 (median) on day 1 had significantly lower 90-day survival than those with ventilatory ratio ≤ 2; they also found VR on day 1 to be significantly associated with 28-day mortality [6, 7].

A recent study in patients who received MV at ICU admission and for a further three days found that a high VR and an increase in VR at day 3 were associated with mortality in those with COVID-19 [8].

The measurement of VR has generated great interest thanks to the ease of application of its formula and the importance of its measurement. It provides relevant information on the dead space fraction (Vd/Vt) and can help to prepare corrective measures to counteract the harmful effects associated with the increase in this parameter.

The aim of the present study was the main objective is to establish that values of pulmonary mechanics, including the ventilatory ratio, are risk factors for 30-day mortality in mechanically ventilated with ARDS due covid-19.

## Materials and methods

A multicenter, observational, retrospective study that included patients with ARDS due to COVID-19 infection admitted to three institutions in the city of Bogota. Consecutive patients from these institutions were included retrospectively by reviewing medical records. Three investigators collected and stored the data independently on a controlled form. The results were presented in accordance with the STROBE guidelines for reporting observational studies in epidemiology [9]. The present study was approved by the Ethics and Research Committee of the Fundación Universitaria Sanitas - CEIFUS 3347-22.

### Patients

**Fig. 1:**
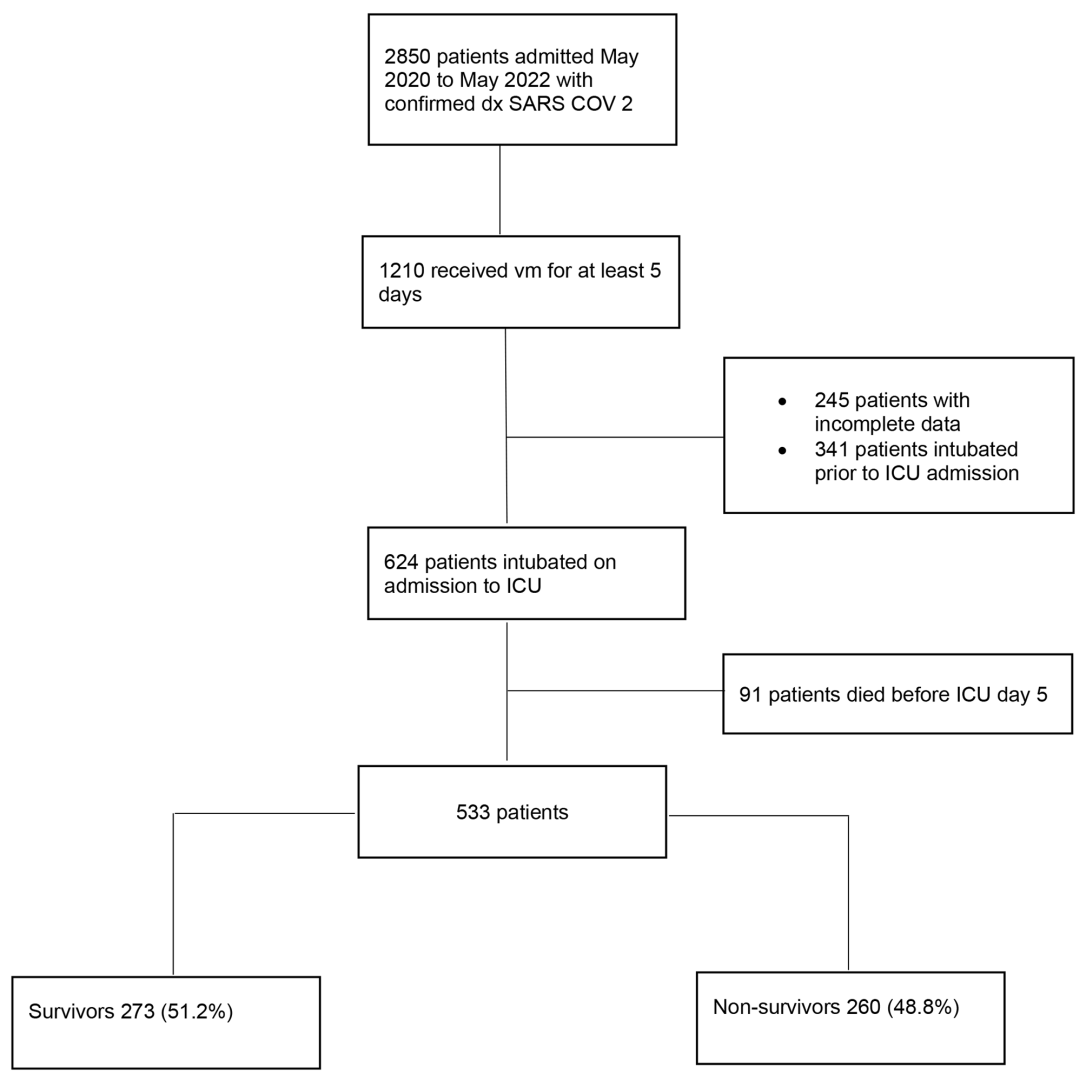
Flowchart of patient screening and enrollment

Consecutive patients admitted to three intensive care units in the city of Bogota with a confirmed diagnosis of COVID-19 between May 2020 and May 2022 were eligible for inclusion in the study. Specific inclusion criteria were: age over 18 years, requirement of MV and an ICU stay of at least 5 days at the Clínica Reina Sofía, Clínica Universitaria Colombia and Clínica Santa María del Lago in the city of Bogotá Colombia. Exclusion criteria were noninvasive MV and/or high-flow nasal cannula, Sequential Organ Failure Assessment Score (SOFA) score above 12 in the first 24 h of ICU admission, requirement of extracorporeal membrane oxygenation (ECMO) in the first 5 days of MV, and incomplete data in the clinical history records (Fig. 1).

### Definitions

ARDS was diagnosed according to the Berlin definition guidelines. Tidal volume was reported in mL/kg of predicted body weight (PBW), the formula used to calculate the predicted or ideal weight was: men height (cm) − 152.4 × 0.91 + 50 women height (cm) − 152.4 × 0.91 + 45.5. Crs was calculated as tidal volume/ (plateau pressure − PEEP). Driving pressure was defined as plateau pressure minus PEEP. Ventilatory ratio was defined as (minute ventilation x PaCO2) / (PBW × 100 × 37.5). Delta measurements were calculated using the difference between the day 3 MV value and the day 1 MV value. Pulmonary mechanics was measured in VCV mechanical ventilation mode.

### Outcome

The primary outcome is mortality at day 30. We also collected data on the duration of the MV.

### Data collection

The following information was recorded: age, sex, predicted weight, severity of the disease assessed with the SOFA scale on ICU admission, comorbidities including diabetes, arterial hypertension, chronic kidney disease, obesity, cardiovascular disease, hypothyroidism, and chronic obstructive pulmonary disease (COPD). The values of the mechanical ventilator programming parameters at day 1 and day 5 were recorded, including PEEP, F_I_O_2_, tidal volume, respiratory frequency, and values of pulmonary mechanics, driving pressure, static compliance and plateau pressure, arterial blood gas values pH, arterial CO_2_ pressure, oxygenation evaluated by the PaO_2_/F_I_O_2_ index, patient position and ventilation efficiency assessed by the VR. The time spent on MV, length of ICU stay, total length of hospital stay and death at 30 days were also included.

### Statistical analysis

Statistical analysis was performed with the statistical software program STATA version 15 licensed to Unisanitas. Categorical variables were described using absolute and relative frequencies, and quantitative variables using measures of central tendency and dispersion, depending on the distribution of the data evaluated by the Shapiro-Wilk test (p < 0.05). Categorical variables were compared by the Chi-square test or Fisher’s exact test, while continuous variables were compared by the nonparametric Wilcoxon rank sum test.

A logistic regression model was carried out after determining a priori a list of possible factors based on their clinical relevance and the results of the bivariate analyses. The backwards method was used to enter the cofactors, and for the final model a p value < 0.05 was considered statistically significant. The odds ratio (OR) and 95% confidence interval (95% CI) were calculated. To evaluate the relevant cofactors, the model’s goodness-of-fit was evaluated with the Hosmer-Lemeshow test. The relative quality of the model was evaluated by calculating the Akaike information criterion (AIC) and the Bayesian information criterion (BIC). The difference in absolute values between days 1 and 5 of the variables associated with mortality was calculated.

Simple collinearity was assessed using Pearson’s correlation coefficient (r). Correlation between the VR and the other study variables was performed using the Spearman correlation coefficient, multicollinearity was assessed by analysing variance inflation factors.

## Results

### Study population

A total of 533 patients were included. Most were male (68.5% n = 365) and the median age was 63 years (53–72). The most frequent comorbidity was arterial hypertension, present in 215 (40.3%), and only 40 (7.5%) had chronic kidney disease. The 30-day mortality rate was 48.8% (260 patients). Table 1 summarizes the main demographic and baseline clinical characteristics of the study population.

**Table 1:** **Baseline demographic and clinical characteristics of patients who received invasive mechanical ventilation on day 1 (IMV)**. Categorical variables are expressed as numbers (percentages) and continuous variables are expressed as medians (interquartile range). p values marked in bold indicate numbers that are statistically significant. **BMI**: body mass index **SOFA**: Sequential Organ Failure Assessment score **MV**: mechanical ventilation **PaCO**_**2**_: carbon dioxide blood pressure **F**_**I**_**O**_**2**_: inspired oxygen fraction **PEEP**: positive end-expiratory pressure **PBW**: predicted body weight **PaO**_**2**_**/F**_**I**_**O**_**2**_: arterial oxygen pressure/inspired oxygen fraction index

VARIABLE	All patients	Survivors	Non-survivors	p value
Age (years)	63 (53–72)	60 (51–67)	66 (57–76)	**0.001**
sex male	365(68.5%)	168 (46%)	197 (54%)	0.93
**Age, categories**				
≤ 50	98	61 (62.2%)	37 (37.8%)	**0.001**
51–60	127	75 (59.1%)	52 (40.9%)	**0.001**
61–70	142	64 (45.1%)	78 (54.9%)	**0.001**
71–80	121	36 (29.8%)	85 (70.2%)	**0.001**
> 80	45	10 (22.2%)	35 (77.8%)	**0.001**
BMI, kg/m^2^	27.6 (25–31)	28 (23–33)	27 (22–32)	0.27
**Comorbidities**				
Hypertension	215	95 (44.2%)	120 (55.8%)	0.45
Dyslipidemia	52	29 (55.8%)	23 (44.2%)	0.14
Diabetes mellitus	129	56 (43.4%)	73 (56.6%)	0.47
Chronic kidney disease	40	14 (35%)	26 (65%)	0.14
Chronic respiratory disease	43	16 (37.2%)	27 (62.8%)	0.22
Chronic cardiac failure	67	21 (31.3%)	46 (68.7%)	**0.009**
Hypothyroidism	80	32 (40%)	48 (60%)	0.23
Obesity	208	105 (50.5%)	103 (49.5%)	0.1
SOFA score	6 (5–7)	6 (5–7)	6 (5–7)	0.15
Days of MV	13 (9–23)	15 (9–28)	13 (8–20)	**0.001**
**Arterial blood gas analysis on admission to ICU**				
**PaO**_**2**_**/F**_**I**_**O**_**2**_ **ratio categories**				
PaO_2_/F_I_O_2_ ratio	122 (89–160)	131 (102–170)	114 (81–156)	0.24
PaO_2_/F_I_O_2_ ratio < 100 mmHg	176	58 (33%)	118 (67%)	
PaO_2_/F_I_O_2_ ratio ≥ 100 and < 200 mmHg	319	164 (51.4%)	155 (48.6%)	
PaO_2_/F_I_O_2_ ratio ≥ 200 and < 300 mmHg	37	23 (62.2%)	14 (37.8%)	
PaO_2_/F_I_O_2_ ratio ≥ 300 mmHg	1	1 (100%)	0 (0%)	
PH	7.31 (7.24–7.36)	7.32 (7.26–7.37)	7.28 (7.21–7.35)	0.18
Lactate, mg/dL	1.5 (1.3–1.85)	1,5 (1.3–1.8)	1.6 (1.3-2.0)	0.47
PaCO_2_, mmHg	49.8 (42–59)	48 (42–57)	51 (43–61)	0.36
**Ventilatory parameters and pulmonary mechanics at the start of MV**				
Tidal volume/PBW (mL/kg)	7.33 (6.87–7.93)	7,2 (6.9–7.8)	7.5 (6.9-8.0)	0.22
Respiratory rate, bpm	18 (18–20)	18 (17–20)	18 (18–20)	0.58
F_I_O_2_, %	60% (50–80)	60% (40–80)	70% (50–90)	**0.02**
PEEP, cmH2O	12 (10–12)	12 (10–12)	12 (10–12)	0.051
Plateau pressure, cmH2O	24 (22–27)	24 (21–26)	24 (22–27)	0.35
Driving pressure, cmH2O	12 (10–15)	12 (10–15)	12 (10–15)	0.15
Compliance, mL/cmH2O	37 (30–45)	38 (31–45)	37 (29–45)	0.41
Ventilatory ratio	1.8 (1.5–2.2)	1.8 (1.4–2.1)	1.9 (1.6–2.4)	0.35
**Position**				
Prone	344 (64.6%)	176 (51.1%)	168 (48.8%)	**0.03**
Supine	189 (35.4%)	70 (37.1%)	119 (62.9%)	

### Clinical features at the start of mechanical ventilation

At the start of MV the median PaO_2_/F_I_O_2_ index was 122 (89–160) mmHg; pH value 7.31 (7.24–7.36); lactate level 1.5 (1.3–1.85) mg/dl; PaCO_2_ 49.8 (42–59) mmHg; with a respiratory rate of 18 [18–20] per minute; tidal volume 7.3 (6.8–7.9) ml/kg ideal weight. Three hundred and forty-four patients (64.5%) required pronation on the first day; driving pressure was 12 [10–15] mmHg, plateau pressure 24 [22–27] mmHg; pulmonary compliance 37 (30–45) ml/cmH_2_O and VR 1.83 (1.48–2.2); median duration of MV was 13 days; in non-survivors median duration of MV was 13 days [8–20] vs. 15 [9–28] in survivors (p 0.001); median F_i_O_2_ in non-survivors was 70% (50–90) vs. 60% (40–80) in survivors p 0.02; SOFA score was 6.0 (5.0–7.0).

### Clinical evolution at day 5 of mechanical ventilation

**Fig. 2:**
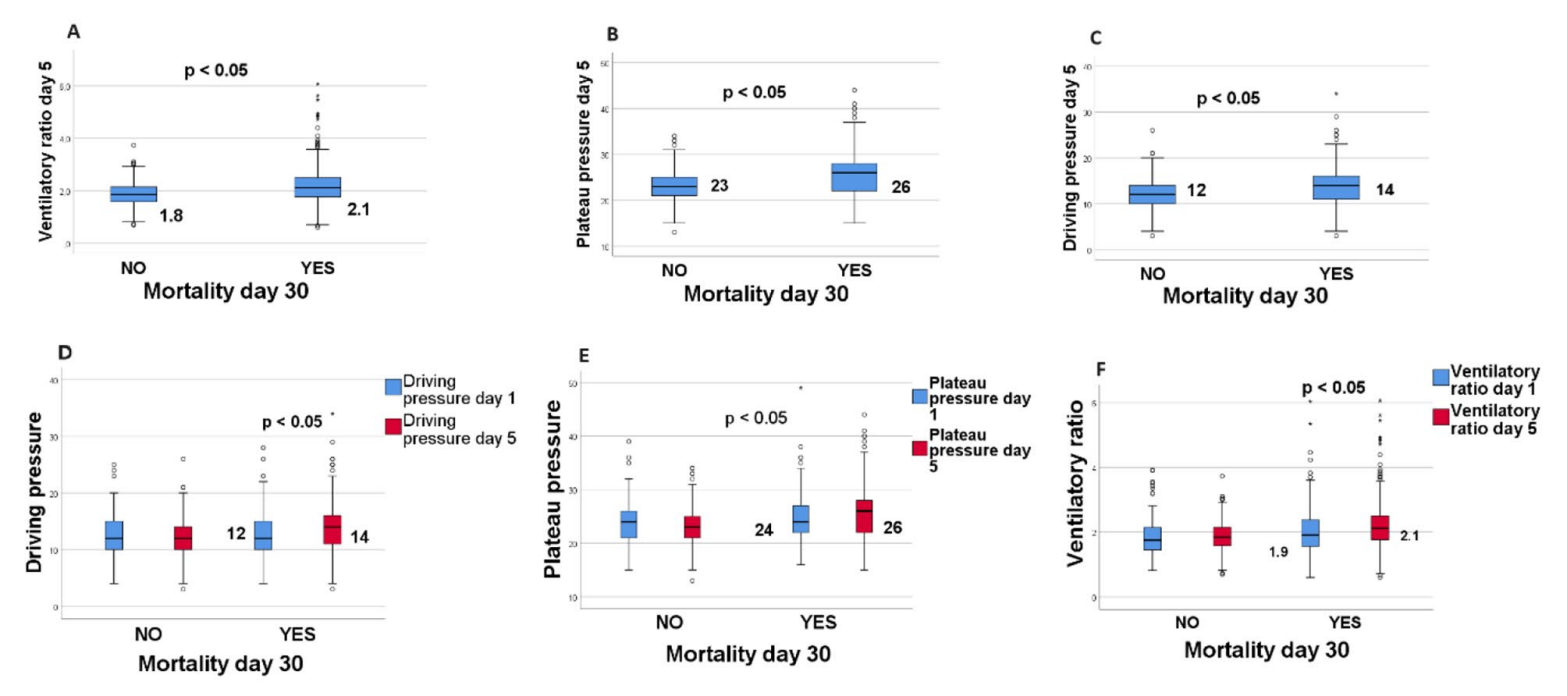
**Association between the different variables measured at day 5 and mortality and changes between the start of mechanical ventilation and day 5**. Association between ventilatory ratio at day 5 (**A**), plateau pressure day 5 (**B**) and driving pressure day 5 (**C**) and mortality at day 30. Figure (**D**) shows the change in the value of driving pressure, (**E**) plateau pressure and (**F**) ventilatory ratio between day 1 and day 5 with statistically significant differences. The horizontal lines of the box-and-whisker plots refer to the median, while the upper and lower lines represent the interquartile range

The main findings found at day 5 are reported in Table 2. There was evidence of an increase in the PaO_2_/F_I_O_2_ ratio to 148 mmHg compared with the start of MV. Non-survivors obtained a lower PaO_2_/F_I_O_2_ index than survivors: 130 (90–159) vs. 169 (140–197) p 0.015, and a lower pH (7.35, range 7.26-7. 41) vs. 7.40 (range 7.37–7.44) p 0.001; plateau pressure was higher in non-survivors (26, range 22–28) vs. 23 [21–25] p 0.001, driving pressure was significantly higher in non-survivors (14, range 11–16) vs. 12 [10–14] p 0.02, and finally VR was significantly higher in non-survivors, 2.1 (1.8–2.5) vs. 1.8 (1.6–2.1) p 0.01 (see Fig. 2).

**Table 2:** **Clinical and respiratory characteristics at day 5 of patients who received invasive mechanical ventilation (IMV)**. Categorical variables are expressed as numbers (percentages) and continuous variables are expressed as medians (interquartile range). p values marked in bold indicate numbers that are statistically significant. **MV**: mechanical ventilation **PaCO**_**2**_: carbon dioxide blood pressure **F**_**I**_**O**_**2**_: inspired oxygen fraction **PEEP**: positive end-expiratory pressure **PBW**: predicted body weight **PaO**_**2**_**/F**_**I**_**O**_**2**_: arterial oxygen pressure/inspired oxygen fraction index

Variable	All patients	Survivors	Non-survivors	p value
Arterial blood gas analysis on day 5 of mechanical ventilation				
PaO_2_/F_I_O_2_ ratio categories				
**PaO**_**2**_**/F**_**I**_**O**_**2**_ **ratio**	148 (109–181)	169 (140–197)	130 (90–159)	**0.015**
PaO_2_/F_I_O_2_ ratio < 100 mmHg	111	58 (33%)	118 (67%)	
PaO_2_/F_I_O_2_ ratio ≥ 100 and < 200 mmHg	348	164 (51.4%)	155 (48.6%)	
PaO_2_/F_I_O_2_ ratio ≥ 200 and < 300 mmHg	72	23 (62.2%)	14 (37.8%)	
PaO_2_/F_I_O_2_ ratio ≥ 300 mmHg	2	2 (100%)	0 (0%)	
PH	7.38 (7.32–7.43)	7.40 (7.37–7.44)	7.35 (7.26–7.41)	**0.001**
Lactate, mg/dL	1.5 (1.2-2.0)	1,5 (1.2-2.0)	1.5 (1.2-2.0)	0.298
PaCO_2_, mmHg	49 (44–57)	47 (42–53)	52 (45–60)	0.103
**Ventilatory parameters and pulmonary mechanics at day 5 of MV.**				
Tidal volume/PBW (ml/kg)	7.6 (7.1–8.2)	7.5 (7.0-7.9)	7.7 (7.1–8.3)	0.217
Respiratory rate, bpm	20 (18–22)	20 (18–20)	20 (18–22)	**0.006**
F_I_O_2_, %	45% (40–60)	40% (40–50)	50% (40–70)	**0.001**
PEEP, cmH_2_O	12 (10–12)	12 (10–12)	12 (11–13)	**0.003**
Plateau pressure, cmH_2_O	24 (22–27)	23 (21–25)	26 (22–28)	**0.001**
Driving pressure, cmH_2_O	13 (10–15)	12 (10–14)	14 (11–16)	**0.02**
Compliance, ml/cmH_2_O	36 (29–46)	38.7 (32.5–46.9)	33.5 (27.2–44.4)	0.25
Ventilatory ratio	2.0 (1.7–2.4)	1.8 (1.6–2.1)	2.1 (1.8–2.5)	**0.01**
**Position**				
Prone	303(56.9%)	123 (40.6%)	180 (59.4%)	**0.001**
Supine	230 (43.1%)	123 (53.5%)	107 (46.5%)	

### Predictors of 30-day mortality

The 30-day mortality rate was 48.8%. The highest mortality was found in the 71–80 years age group in which 85 patients died (70.2% of the total for the age group). The mortality rate was 68.6% in men vs. 31.4% in women. The pronation was a protective factor for mortality (OR crude 0.77. 95% CI 0.66–0.90 p 0.001) after including possible confounding factors such as days of MV, initial lactate, PaO_2_/F_I_O_2_ index at day 5, ventilatory ratio at day 5, plateau pressure at day 5, driving pressure at day 5 and age, the logistic regression model showed that the VR was independently associated with mortality (OR 2.1, 95% CI 1.35–3.3 p 0.001). As secondary findings, driving pressure (OR 2.9, 95% CI 1.7-5.0 p 0.001), plateau pressure (OR 2.0, 95% CI 1.02-4.0 p 0.04) and age range 71–80 (OR 5.2, 95% IC 2.76-10.0 p 0.001). Were independently associated with mortality. (see Table 3).

**Table 3:** **Multivariate model assessing predictors of mortality day 30**. **R**^**2**^**33%** Mixed effects model considering a binomial distribution **MV**: mechanical ventilation, **IC 95%**: confidence interval at 95% **PaO**_**2**_**/F**_**I**_**O**_**2**_: arterial oxygen pressure/inspired oxygen fraction index p values marked in bold are statistically significant

Mortality	Odds ratio	(95% CI)	p value
Days of MV	0.91	0.88–0.93	**0.001**
Initial lactate	1.38	1.03–1.86	**0.016**
PaO_2_/F_I_O_2_ day 5	0.98	0.97–0.98	**0.001**
Ventilatory ratio day 5	2.1	1.35–3.3	**0.001**
Driving pressure	2.9	1.70-5.0	**0.001**
Plateau pressure	2.0	1.02-4.0	**0.04**
Age			
51–60	1.56	0.85–2.87	0.14
61–70	1.94	1.08–3.48	**0.02**
71–80	5.2	2.76-10.0	**0.001**
> 80	6.5	2.68–15.8	**0.001**

The increases in VR at day 5 above 2.0 (OR 1.42 95% CI 1.03–2.01, p 0.04), in driving pressure at day 5 (OR 1.56, 95% CI 1.10–2.22, p 0.001) and in plateau pressure at day 5 (OR 1.9 95% CI 1.34–2.69, p 0.001) significantly raised the risk of mortality, but the deterioration of the PaO_2_/F_I_O2 index was not associated with an increased mortality risk (Table 4).

**Table 4:** **Bivariate model of change between day 1 and 5 of MV**. **PaO**_**2**_**/F**_**I**_**O**_**2**_: arterial oxygen pressure/inspired oxygen fraction index. **CI**: confidence interval. **Delta ventilatory ratio**: ventilatory day 5 - ventilatory ratio day 1. **Delta driving pressure**: driving pressure day 5 - driving pressure day 1. **Delta plateau pressure**: plateau pressure day 5 - plateau pressure day 1. **Delta PaO**_**2**_**/F**_**I**_**O**_**2**_: PaO_2_ /F_I_O_2_ day 5 - PaO_2_ /F_I_O_2_ day 1

Variable	Odds ratio	(95% CI)	p value
Delta ventilatory ratio	1.42	(1.03–2.01)	0.04
Delta driving pressure	1.56	(1.10–2.22)	0.01
Delta plateau pressure	1.9	(1.34–2.69)	0.001
Delta PaO_2_/F _I_O _2_	1.34	(0.96–1.56)	0.051

## Discussion

The aim of the present study was the main objective is to establish that values of pulmonary mechanics, including the ventilatory ratio, are risk factors for 30-day mortality in mechanically ventilated with ARDS due covid-19. The findings show that the VR, driving pressure, plateau pressure measured at day 5 and the change in these variables between days 1 and 5, age and heart failure were associated with mortality at 30 days of follow-up. The 30-day mortality rate in patients who remained on MV for at least 5 days was 48.8%.

The demographic variables associated with higher mortality were age, in agreement with several recent studies [10–12], heart failure and male sex [11, 13, 14].

In agreement with previous studies [8, 15–17], we did not find an association between the PaO_2_/F_I_O_2_ index and mortality at the beginning of MV, and nor did oxygenation impairment from day 1 to day 5 appear to be associated with mortality. In this cohort of patients the median compliance was 36 ml/cmH_2_O, also in agreement with several other studies in patients with ARDS (18, 19, 20, 21); however, compliance was not significantly associated with prognosis. It has been proposed that ARDS patients with COVID-19 may have two different phenotypes related to pulmonary compliance and that this distinction could be used to guide a rigorous, personalized titration of the PEEP value [22]. In our study, however, the PEEP value did not differ significantly between survivors and non-survivors measured at the different time points.

Among the complementary treatments for refractory hypoxemia, prone position was used in 56.9% of the patients considered to present relevant values. The pronation reduced the risk of mortality, as has been reported elsewhere [23]. We did not obtain data on the specific causes of nonpronation. In this cohort driving pressure was shown to be a relevant variable and its increase was associated with mortality, as other investigations have shown [24–26]. Also in agreement with other studies [23, 27], the increase in plateau pressure meant a higher risk of mortality.

The VR is a validated index in controlled modes of MV. It is frequently used, given the ease of its calculation at the patient’s bedside by recording the PaCO_2_ and minute ventilation, and it can be used as a surrogate for the dead space fraction vd/vt; a value close to 1 means that pulmonary ventilation is normal [4]. Deficient ventilation is frequent in patients with ARDS, as previous studies have reported [28–30]. High VR values in patients with ARDS without COVID-19 were associated with mortality [15, 31]; additionally, it has been demonstrated that patients with ARDS and COVID-19 presented a high VR associated with increased vd/vt [21, 32], as we found in our study.

We did not find an association between VR at day 1 and 30-day mortality. In previous work, this association was found to be statistically significant at the beginning of MV [8, 15]. In contrast to our results, in a cohort of 927 consecutive ARDS patients with COVID-19 it was reported that a rising VR at day 3 was not independently associated with 28-day mortality after adjustment for a baseline risk model that included chronic comorbidities and ventilatory and oxygenation parameters [16]. A major difference between that study and ours is that we evaluated the change between day 1 and day 5, finding that the increase of this value above 2.0 on day 5 was associated with a greater risk of mortality at 30 days, this finding can be explained by the deterioration of pulmonary mechanics during the course of the disease.

In our cohort there were no significant differences in the SOFA scale measured on day 1 between survivors and non-survivors; this may probably be due to the performance of the scale used early in the ICU, Another aspect to take into account and which may justify this finding is that the clinical condition of the patients at the beginning of the MV did not show high severity and later the function of the organs could deteriorate rapidly in this group of patients, considering the moment of evaluation of this severity scale in the ICU as a relevant topic for future research.

Among the limitations of the study, we should note that measurements on day 1 and day 5 are only two time points in the course of ARDS due to COVID-19 and may represent the moments when the disease was at its worst in the ICU. Nor was information on metabolism and CO_2_ production available, two phenomena that may intervene in ventilatory efficiency, is a topic that has been previously researched; finally, the retrospective nature of the study may have introduced biases.

In addition, we were unable to calculate the sample size objectively, although the number of participants we obtained sufficient power to detect significant differences in the primary outcome. Strengths of the study include the multicenter design that allows the collection of information from other institutions, the multivariate analysis at day 1 and day 5 and the evaluation of the change produced at these two time points that substantiate to the results obtained.

The results of this study have implications for clinical practice, since they show that the measurement of the VR is a tool that can be added to driving pressure and plateau pressure for the prognosis of ARDS patients with COVID-19, thanks to its simple formula that can be applied at the patient’s bedside to estimate the dead space fraction. The findings presented here may help to guide decision making for ARDS patients on MV in the ICU.

## Conclusions

Ventilatory ratio, plateau pressure, driving pressure, and age were identified as independent risk factors for 30-day mortality in patients with ARDS due to COVID-19 on day 5 of invasive mechanical ventilation.

## Data Availability

The datasets used and/or analysed during the current study are available from the corresponding author on reasonable request.

## Abbreviations

ICU: Intensive care unit
ARDS: Acute Respiratory Distress Syndrome
COVID 19: Coronavirus disease 2019
PEEP: Positive end-expiratory pressure
VR: Ventilatory ratio
F_I_O_2_: Fraction of inspired oxygen
PaCO_2_: Partial pressure of carbon dioxide in arterial blood
VD/VT: Dead space fraction
SOFA: Sequential Organ Failure Assessment score
PaO_2_/F_I_O_2_: Arterial oxygen pressure/inspired oxygen fraction index
STROBE: Strengthening the reporting of observational studies in epidemiology
ECMO: Extracorporeal membrane oxygenation
Crs: Static compliance
